# Incidence of Thrombosis in COVID-19 Patients Compared to Non-COVID-19 Sepsis Patients in the Intensive Care Unit

**DOI:** 10.3390/jcm13102974

**Published:** 2024-05-18

**Authors:** Sherri Huang, Ashley Perry, Carlos Sanchez Parra, Adriana Gonzalez Torriente, Haider Ghumman, Shaun Charkowick, Joshua Colon, McKenzi Heide, Michael Jaglal, Rahul Mhaskar, Juan Felipe Rico

**Affiliations:** 1 Department of Internal Medicine and Pediatrics, University of South Florida, Tampa, FL 33606, USA; 2 Division of Pediatric Cardiology, Texas Children’s Hospital, Houston, TX 77030, USA; 3 Morsani College of Medicine, University of South Florida, Tampa, FL 33612, USA; 4 Department of Hematology, Morsani College of Medicine, University of South Florida, Tampa, FL 33612, USA; 5 Department of Hematology and Oncology, Moffitt Cancer Center, Tampa, FL 33612, USA; 6 Department of Pediatrics, Morsani College of Medicine, University of South Florida, Tampa, FL 33606, USA

**Keywords:** COVID-19, sepsis, venous thromboembolism, intensive care unit

## Abstract

**Background/Objectives:** The hypercoagulable state associated with COVID-19 infection is associated with adverse outcomes and mortality. Studies have also demonstrated high rates of venous thromboembolism (VTE) events among patients with sepsis. We aimed to evaluate how the increase in thrombotic events in critically ill patients with COVID-19 infection compares to that of critically ill patients with non-COVID-19 sepsis. **Methods:** A chart review was performed of patients 18 years or older admitted to the intensive care unit (ICU) at Tampa General Hospital between 1 January 2020 and 31 December 2020 diagnosed with COVID-19 or sepsis secondary to other pathogens. Non-COVID-19 sepsis patients and COVID-19 patients were propensity-matched 3:1 on the Charlson Comorbidity Index. Multivariate analyses adjusting for confounding were conducted to report odds ratio (OR) and 95% confidence intervals (95% CIs) of predictors for thrombotic events and overall mortality. **Results:** After propensity score matching, 492 sepsis patients and 164 COVID-19 patients were included in the analysis. COVID-19 patients were significantly older (*p* = 0.021) and showed higher BMI (*p* < 0.001) than sepsis patients. COVID-19 patients did not show significantly higher odds of thrombosis after adjustment for confounders (OR 0.85, 95% CI 0.42–1.72), but had significantly lower odds of mortality than sepsis patients (OR 0.33, 95% CI 0.16–0.66). **Conclusions:** Our results suggest that further study is required to lower the rate of VTE in COVID-19 and non-COVID-19 sepsis patients admitted to the ICU; it is also reasonable to consider similar thromboembolism practices between these two patient groups.

## 1. Introduction

The COVID-19 pandemic to date is responsible for 6.98 million deaths and has devastated economies and healthcare infrastructure worldwide [1]. The medical community has gained an understanding of the pathophysiology and long-term effects of COVID-19, yet much remains unknown. Although COVID-19 is a part of the previously established family of single positive-strand RNA coronaviruses, it causes more severe disease than the common cold. Severe complications of COVID-19 include acute respiratory distress syndrome (ARDS), venous thromboembolism (VTE), and multiple organ dysfunction [2,3]. In mid-January 2021, more than 28,000 COVID-19 patients were admitted to intensive care units (ICUs) at a given time [4]. For hospital infrastructure, the local burden of COVID-19 warrants regular triage of hospital beds for COVID-19 patients, with ongoing research into viral pathogenesis for the prognostic stratification of patient outcomes and improving management guidelines.

The pathogenesis of COVID-19 involves three general stages: viral replication, immune hyperactivity, and pulmonary destruction [2,5]. The virus has been shown to have tropism for the pulmonary system, utilizing angiotensin-converting enzyme-2 (ACE2) to direct its entry into cells [2,6,7]. Upon replication, the virus multiplies and upregulates the immune system by promoting the secretion of Type I interferons in addition to proinflammatory cytokines and chemokines [8,9,10]. The systemic activation of coagulation is a part of the pathophysiology of COVID-19. It is associated with adverse outcomes and mortality, and numerous studies have demonstrated the hypercoagulable state associated with COVID-19 infection. In addition to comorbidities and advanced age, elevated D-dimer levels in COVID-19 patients have increased the risk of in-hospital mortality [11]. The rate of VTE in COVID-19 patients across studies is approximately 20% [12]. The small vessels and capillaries of deceased COVID-19 patients revealed fibrin thrombi and extensive fibrin deposition [13]. Compared to the lungs of H1N1 ARDS patients, the lungs of COVID-19 patients demonstrated distinctive vascular features, including widespread thrombosis [14]. Consistent with these pathological findings, D-dimer levels are substantially higher in patients with severe COVID-19 disease, i.e., those needing mechanical ventilation or ICU admission, or those who died, compared to those with a mild infection [15]. Furthermore, disseminated intravascular coagulation (DIC) occurred in over 70% of hospitalized patients who succumbed to COVID-19 compared to <1% in those who survived [16]. Although anticoagulation therapy is widely utilized to prevent VTE occurrence, the cumulative incidence of VTE in COVID-19 ICU patients was nevertheless found to be 31% [17]. Together, these observations suggest that the hypercoagulable state seen in COVID-19 patients is an important component of the pathophysiology of COVID-19 disease and may be associated with adverse outcomes and mortality.

Historically, studies have also demonstrated high rates of thrombotic events among patients with sepsis. A multicenter prospective study by Kaplan et al. found that 37.2% of patients with severe sepsis and septic shock developed VTE [18]. This rate appears to be similar to a meta-analysis conducted by Porfidia et al., which showed a VTE rate of approximately 26% in patients with COVID-19 [19]. Thrombosis in sepsis is thought to result from a complex interplay of numerous factors, from systemic inflammation, endothelial injury, altered blood flow, and hypercoagulability to fibrinolytic dysregulation [20,21,22]. Sepsis results in endothelial damage, eventually leading to the activation of platelets that then recruit leukocytes, leading to the formation of platelet–leukocyte aggregates that promotes thrombus formation [21]. Sepsis also triggers the systemic release of procoagulant molecules such as tissue factor, leading to widespread activation of the extrinsic coagulation cascade. This in turn results in the rapid conversion of prothrombin to thrombin, which drives fibrin clot formation [20,21]. Finally, sepsis impairs the regulation of fibrinolysis, allowing for excessive fibrin accumulation and thrombus formation [22]. This may occur through several mechanisms, including the decreased production of proteases involved in fibrinolysis or the increased expression of inhibitors of fibrinolysis. Complement activation during sepsis also leads to the generation of prothrombotic mediators, such as C3a and C5a, which promote neutrophil and monocyte activation and recruitment to the site of thrombus formation; these cells release procoagulant molecules and proteases involved in fibrinolysis [23].

Whether there is an increase in thrombotic events as a result of COVID-19-specific infection or a general severe infection response is unknown. Prior studies have evaluated the different possible mechanisms of hypercoagulability in patients with COVID-19, including the effect of COVID-19 on platelet aggregation, the upregulation of coagulation factors, and the downregulation of anti-thrombotic proteins [24,25,26]. Nevertheless, definitive consensus is lacking on whether COVID-19 hypercoagulability arises from a COVID-19-specific mechanism. Previous studies have found increased rates of coagulation parameters when comparing severe to mild COVID-19 disease but not when comparing COVID-19 to non-COVID-19 infection or when limited in sample size [27,28]. Comparing the rates of VTE between COVID-19 sepsis and non-COVID-19 sepsis is important because doing so would allow us to determine whether COVID-19 sepsis carries a unique risk of thrombosis compared to other forms of sepsis. If COVID-19 sepsis is found to have higher VTE rates than non-COVID-19 sepsis, this would suggest specific pathways of the viral infection or host response that contribute to an increased risk of thrombosis in COVID-19 infection, rather than sepsis-triggered pathways. That is, different VTE rates may indicate COVID-19-specific mechanisms warranting further investigation. Such information would be critical for developing personalized treatment plans for patients with COVID-19 sepsis.

Conversely, if the rates of VTE are similar between COVID-19 sepsis and non-COVID-19 sepsis, this would suggest that sepsis rather than specifically the COVID-19 virus itself is a primary driver of thrombosis. This would be valuable knowledge, as it would mean that treatment strategies for thrombosis should be focused on managing the systemic inflammation and endothelial dysfunction that accompany sepsis. For example, in the escalation of care to the ICU, a decision for VTE prophylaxis versus treatment is still a point of ongoing synthesis and guideline updates. Understanding the relative contribution of VTE in COVID-19 patients helps risk-stratify and potentially clarify thromboembolism management from sepsis guidelines. Comparable VTE rates would also emphasize the importance of early identification and treatment of sepsis, regardless of the underlying cause.

To delineate the role of VTE in COVID-19 ICU admission and outcomes, we compared the rate of thrombotic events across patients with COVID-19 disease and all patients with sepsis or septic shock.

## 2. Materials and Methods

This retrospective cohort study was performed using a review of the electronic medical records from 1 January 2020 to 31 December 2020 of patients in the intensive care unit at Tampa General Hospital. All collected data was anonymized. This study was reviewed and deemed exempt by our Institutional Review Board (IRB) at the University of South Florida (Study #001930 meets criteria for exemption from IRB review). The targeted sample size was determined using the 26% rate of VTE in all COVID-19 patients in the above meta-analysis. For sepsis, we used the 32% rate noted in critically ill patients. The rate of VTE in hospitalized sepsis patients is not currently known. The inclusion criteria of patients were intensive care unit admission, age greater than 18 years, and diagnosis of sepsis or COVID-19 infection.

Patients were identified as diagnosed with COVID-19 and/or sepsis by ICD-10 codes. At the institution in which this study took place, the ICU admission criteria include criteria identifying the patient as severely ill and/or requiring management that cannot take place on the floor or in the step-down unit. These ICU admission criteria include the need for frequent vitals monitoring; increased or advanced respiratory support such as heated high-flow nasal cannula above 60 L/fiO_2_ 60%, BiPAP, or mechanical intubation; need for sedation; central line placement; and/or pressors. The risk of decompensation is another criterion for which providers employ established guidelines alongside clinical judgment, which may include an assessment of the patient’s labs, need for escalation of oxygen, and comorbidities.

COVID-19 infection was defined as having a positive SARS-COV-2 reverse-transcriptase polymerase chain reaction test by nasopharyngeal/oropharyngeal swab or sputum specimen.

There were no exclusion criteria.

### 2.1. Data Collection

The following patient-specific information was recorded: demographics (age and sex); relevant comorbidities, including risk factors for DVT (see below) and variables required for calculation of the Charlson Comorbidity Index (CCI); need for hospital admission; need for endotracheal intubation; hospital length of stay; completion of hospitalization (discharge, disposition, or death); type of infection, if known; bleeding events; arterial and venous thrombotic events; end-organ injury or failure, presence of acute respiratory distress syndrome; anticoagulation administered; antiplatelet agents; antihistamine use; coagulation; inflammatory laboratory parameters (WBC, platelets, ESR, procalcitonin, fibrinogen, D-dimer, ferritin, lupus anticoagulant, anti-phospholipid antibodies); PT/PTT; and renal and liver function tests. Cardiac markers and assessments such as troponins, CPK, BNP, and echocardiogram and EKG results, where available, were also collected. The initial, highest, and lowest values were recorded for laboratory tests.

DVT risk factors were identified as age > 60, BMI > 30, having a central venous line, immobilization, active cancer, recent trauma or surgery < 1 month ago, heart failure, respiratory failure, acute myocardial infarction or stroke, any ongoing hormonal treatment, any active rheumatologic disorder, diabetes, prior VTE, and thrombophilia.

### 2.2. Statistical Analysis

Non-COVID-19 sepsis patients and COVID-19 patients who were admitted to the ICU were propensity-matched 3:1 on the CCI. Differences in patient characteristics between sepsis and COVID-19 patients were assessed using chi-square tests for categorical variables and Mann–Whitney U tests for continuous variables. The multivariate analysis consisted of two binary logistic regression models for thrombosis diagnosis and mortality. Variables for multivariate analysis were chosen based on the significance in the univariate analysis of thrombosis diagnoses and data availability. Variables with over 35% of data missing were not included in the final multivariate models. A significance level of *p* < 0.05 was utilized. Analysis was completed using SPSS Version 27.

## 3. Results

After propensity score matching on the CCI, 656 patients were included, with a 3:1 ratio of 492 non-COVID-19 sepsis patients and 164 COVID-19 patients. Comparisons of patient characteristics between the two groups are shown in Table 1. COVID-19 patients were significantly older (*p* = 0.021) and had higher BMI (*p* < 0.001) than non-COVID-19 sepsis patients. COVID-19 patients had lower proportions of active cancer (*p* = 0.001), organ failure (*p* = 0.003), and antihistamines administered (*p* < 0.001). COVID-19 patients had higher proportions of diabetes (*p* < 0.001), steroids administered (*p* = 0.013), and anticoagulation administered (*p* < 0.001). Non-COVID-19 sepsis patients had a longer length of stay on average (23.28 ± 36.72 days for non-COVID-19 sepsis patients and 14.29 ± 15.44 days for COVID-19 patients, *p* < 0.001). Non-COVID-19 sepsis patients had a higher mortality rate than COVID-19 patients (36.8% vs. 22.1%, *p* = 0.003). There were no significant differences in the rates of ongoing hormonal treatment, antiplatelet agents administered, thrombophilia, bleeding events, heart failure diagnosis, AMI/stroke diagnosis, previous VTE, HIV diagnosis, or respiratory failure diagnosis between the COVID-19 and non-COVID-19 sepsis cohorts (Table 1).

There was no significant difference in thrombosis rates among ICU patients with COVID-19-specific infection compared to non-COVID-19 sepsis (17.1% diagnosed with thrombosis among COVID-19 patients and 16.3% among non-COVID-19 sepsis patients, *p* = 0.808). Multivariate analysis of the thrombosis outcome showed significant associations with male sex (OR 1.80, 95% CI 1.03–3.12), age (OR 1.04, 95% CI 1.02–3.12), antiplatelet agents (OR 3.41, 95% CI 2.01–5.81), highest PT value (OR 1.02, 95% CI 1.01–1.04), first ALT value (OR 1.001, 95% CI 1.000–1.002), and a protective relationship of the steroids administered (OR 0.42, 95% CI 0.24–0.71). Compared to non-COVID-19 sepsis patients, COVID-19 patients did not show significantly higher odds of thrombosis after adjustment for these covariates (OR 0.85, 95% CI 0.42–1.72). Details of univariate and multivariate thrombosis models are shown in Table 2. Supplementary Table S1 shows the complete list of laboratory parameters collected.

Overall mortality was also assessed with multivariate analysis using the same covariates with the addition of thrombosis as a predictor. Compared to non-COVID-19 sepsis patients, COVID-19 patients had significantly lower odds of mortality (OR 0.33, 95% CI 0.16–0.66). The diagnosis of a thrombotic event was not a significant predictor of mortality (OR 1.48, 95% CI 0.82–2.66). Significant predictors of mortality included age (OR 1.04, 95% CI 1.02–1.056), steroids administered (OR 2.35, 95% CI 1.47–3.76), first platelet count (OR 0.998, 95% CI 0.996–1.000), highest PT value (OR 1.02, 95% CI 1.001–1.031), and first ALT value (OR 1.002, 95% CI 1.000–1.004).

## 4. Discussion

Our study evaluated VTE rates in ICU-admitted patients and found no significant differences between the rates of VTE in COVID-19 patients compared to non-COVID-19 sepsis patients despite matching of CCI scores. The COVID-19 patients in our sample had lower mortality odds and VTE was not a predictor of mortality. A lower percentage of non-COVID-19 patients received anticoagulation compared to the COVID-19 patients despite having higher rates of active cancer, endotracheal tubes, and central lines, all risk factors for thrombosis. This was despite a similar number of bleeding events in both groups. Perhaps the greater rate of organ failure and medical instability of the non-COVID-19 sepsis patients led to hesitancy or contraindications to anticoagulation, which may increase the rate of VTE in sepsis patients. Also, sepsis patients may have had higher rates of coagulopathy, which was not captured in our sample, although there were no statistically significant differences between a history of previous VTE, thrombophilia, and antiplatelet agents administered between the two cohorts.

Steroid administration was higher in the COVID-19 patients, consistent with current treatment recommendations for severe COVID-19 infections. Interestingly, antihistamine use was higher in non-COVID-19 sepsis patients. Perhaps these patients had higher rates of allergic reactions, nausea, or pruritic reactions to the various medications given while in the ICU.

VTE is a leading cause of morbidity and mortality, and patients with inflammatory conditions are at a higher risk of both initial and recurrent episodes of VTE. Both COVID-19 infection and sepsis are highly inflammatory states. Historically, studies have demonstrated high rates of thrombotic events among patients with sepsis [18]. Patients meeting sepsis criteria in the ICU without an identified presence or history of VTE follow the guidelines for prophylactic dosing of anticoagulation, i.e., the American College of Chest Physicians recommends pharmacologic thromboprophylaxis for all patients admitted to the ICU [29].

The rate of VTE in severely ill COVID-19 patients ranges from about 20–30% across meta-analyses [19,30,31,32]. Our study finding of 17% is, therefore, similar to the rate of VTE in COVID-19 patients across meta-analyses. For COVID-19 patients admitted to the wards compared to ICU, current guidelines recommend therapeutic-dose anticoagulation, which has been demonstrated to reduce mortality and the need for organ support in ward-level patients without significantly increasing bleeding risk [33]. In contrast, in ICU patients, the risk of bleeding outweighs the benefit of therapeutic-dose anticoagulation. As such, for COVID-19 patients admitted to the ICU, current guidelines recommend prophylactic over therapeutic dosing [33]. Consistent with these guidelines, our study findings suggest that the escalation of prophylactic dosing in COVID-19 patients admitted to the ICU is not necessarily warranted and that it is reasonable to consider similar thromboembolism practices between COVID-19 and non-COVID-19 critically ill patients.

In our study, COVID-19-positive patients were identified through standard screening procedures and were not identified or stratified by clinical presentation, such as dyspnea, oxygen saturations, PaO_2_/FiO_2_, or development of respiratory failure, including acute respiratory disease syndrome. Indeed, one limitation of this study is its retrospective aspect which relied on identifying a set of variables to collect and the available data at the time of data collection. As such, we were unable to determine additional clinical characteristics, such as the respiratory requirements for patients in the COVID-19 cohort, other than the binary identification of whether a patient underwent endotracheal intubation. Further evaluation of how COVID-19 clinical infection severity as defined by respiratory impairment correlates with VTE rates can likely assist in determining VTE as a prognostic factor. For example, it is unknown how the increasing severity of COVID-19 respiratory infection correlates with VTE rates and mortality. Indeed, predictors of COVID-19 disease severity and worsened prognosis correlate with inflammatory markers, i.e., D-dimer levels, LDH, and high-sensitivity cardiac troponin I [34,35]. Increased inflammatory states lead to the disruption of the blood vessel interface, creating an environment conducive to hypercoagulability. Although our study compared laboratory parameters including D-dimer, ferritin, and platelet count, we did not collect CRP or LDH.

Furthermore, previous studies have suggested that COVID-19 diagnosis is associated with increased arterial thromboses, a phenomenon largely attributed to the rupture of atherosclerotic plaques followed by platelet aggregation around the lesion [36]. Studies have found that COVID-19 clot histology is more megakaryocyte-rich [37]. Our chart review approach did not capture the characteristics of the thromboembolic events across our cohorts such as the location of thromboses or the method by which the VTE was diagnosed. Differentiating the locations of thromboses between our COVID-19 and non-COVID-19 sepsis cohort may reveal novel pathophysiology, and future studies may both evaluate the location and differentiate between arterial and venous thromboses. However, our findings support the current findings related to VTE rates and anticoagulation guidelines for COVID-19 patients in the ICU and suggest that further exploration of the pathophysiology of thrombotic events in COVID-19 patients can enlighten disease prognosis compared to non-COVID-19 sepsis.

Our study used sepsis ICD codes to identify patients with sepsis. We used institutional guidelines to identify both patients warranting a sepsis ICD code and ICU disposition. While the limitations to this approach include less resolution when stratifying those in severe sepsis or septic shock, our univariate and multivariate analysis accounts for rates of multi-organ failure, markers of inflammation, and markers of organ injury. Future studies comparing VTE rates may evaluate the rates of thrombosis as stratified by the severity of sepsis. Given the retrospective aspect of this study, we were also unable to determine the collection timepoints of patients’ laboratory values relative to admission. With regard to the collection timepoints of parameters such as WBC, platelets, ESR, procalcitonin, PT/PTT, fibrinogen, and ferritin, we captured the range of each patient’s clinical parameters by recording the highest and lowest values along with initial values (Supplementary Table S1). While this approach may not reflect the severity of illness, the compilation of multiple laboratory parameters along with the patient’s past medical history and ICU status serves as a representative measure of overall clinical severity in this study.

In addition, this was a single-institution study, so the prophylactic algorithms for VTE in this sample may differ from other institutions, limiting our study’s generalizability. In addition, not all patients received the same prophylactic regimen for anticoagulation. During the timeframe of our study, it was common for physicians at our institution to use intermediate or treatment-dose anticoagulation rather than prophylactic dosing in COVID-19 patients. Despite this, bleeding rates were similar in the COVID-19 group compared to the sepsis patients. Finally, our study could not look into the factors that led some patients to receive anticoagulation while others did not. Due to this, conclusions regarding the differences in anticoagulation rates between the two groups cannot be made.

In summary, our study found that thrombosis rates were similar between critically ill COVID-19 and non-COVID-19 sepsis patients in the ICU. Mortality was increased in the non-COVID-19 sepsis patients, as were the rates of endotracheal tube use, central lines, organ failure, antihistamine use, and active cancer. Rates of diabetes, obesity, and steroid administration were higher in the COVID-19 group.

## Figures and Tables

**Table 1:** Age, BMI, length of stay (LOS), medical history, and relevant treatments in COVID-19 and non-COVID-19 sepsis patients.

Variable	COVID-19 (N = 164)	Sepsis (N = 492)	*p*-Value
Age	61.60 ± 15.49	58.25 ± 15.86	0.021
BMI	31.81 ± 8.81	29.16 ± 8.89	<0.001
LOS (days)	14.29 ± 15.44	23.28 ± 36.72	<0.001
Endotracheal tube	22 (13.4)	147 (29.9)	<0.001
Central line	31 (18.9)	182 (37.0)	<0.001
Active cancer	6 (3.7)	62 (12.6)	0.001
Steroids administered	93 (56.7)	224 (45.5)	0.013
Anticoagulation administered	122 (74.4)	261 (53.0)	<0.001
Antihistamine agent administered	43 (26.2)	235 (47.8)	<0.001
Diabetes diagnosis	85 (51.8)	162 (32.9)	<0.001
Organ failure diagnosis	117 (71.3)	405 (82.3)	0.003
Mortality	27 (22.1)	151 (36.8)	0.003
Ongoing hormonal tx	1 (0.6)	1 (0.2)	0.438
Antiplatelet agents administered	68 (41.5)	176 (35.8)	0.192
Thrombophilia present	7 (4.3)	18 (3.7)	0.724
Bleeding events	12 (7.3)	62 (12.6)	0.064
Arterial and thrombotic events	72 (43.9)	242 (49.2)	0.241
Heart failure diagnosis	50 (30.5)	166 (33.7)	0.443
AMI/stroke diagnosis	25 (15.2)	108 (22.0)	0.064
Previous VTE	11 (6.7)	51 (10.4)	0.165
HIV diagnosis	3 (1.8)	11 (2.2)	1.000
Respiratory failure diagnosis	83 (50.6)	269 (54.7)	0.366

N (%) or mean ± standard deviation.

**Table 2:** Univariate and multivariate models.

	Outcome: Thrombosis				Outcome: Mortality	
	Univariate		Multivariate		Multivariate	
Covariate	OR (95% CI)	*p*	OR (95% CI)	*p*	OR (95% CI)	*p*
COVID-19 (ref: sepsis)	1.06 (0.66–1.70)	0.808	0.85 (0.42–1.71)	0.640	0.33 (0.16–0.66)	0.002
Sex (ref: female)	0.54 (0.35–0.84)	0.006	1.80 (1.03–3.12)	0.038	0.88 (0.55–1.39)	0.571
Age	1.03 (1.02–1.05)	<0.001	1.04 (1.02–1.06)	<0.001	1.04 (1.02–1.06	<0.001
Steroids administered	0.55 (0.36–0.84)	0.006	0.42 (0.24–0.71)	0.001	2.35 (1.47–3.76)	<0.001
Antiplatelet agents	4.37 (2.82–6.78)	<0.001	3.41 (2.01–5.81)	<0.001	1.10 (0.68–1.78)	0.691
Diabetes	1.54 (1.01–2.33)	0.043	0.92 (0.53–1.58)	<0.001	0.76 (0.47–1.25)	0.282
Platelet count (first)	0.998 (.997–1.00)	0.033	0.998 (0.996–1.001)	0.213	0.998 (0.996–1.000)	0.029
PT (highest)	1.02 (1.01–1.03)	0.004	1.02 (1.01–1.04)	0.002	1.02 (1.001–1.031)	0.031
ALT (first)	1.001 (1.000–1.002)	0.036	1.001 (1.000–1.002)	0.041	1.002 (1.000–1.004)	0.038
Thrombosis	N/A	N/A	N/A	N/A	1.48 (0.82–2.66)	0.192

## Data Availability

The anonymized datasets collected during and/or analyzed during the current study will be made available as open data via an online data repository.

## Supplementary Materials

The following supporting information can be downloaded at: https://www.mdpi.com/article/10.3390/jcm13102974/s1, Table S1: Patient’s Charleston Comorbidity Index (CCI), Age, Length of Stay (LOS), and Initial, Highest and Lowest Lab Values.
